# Telomerase-based GX301 cancer vaccine in patients with metastatic castration-resistant prostate cancer: a randomized phase II trial

**DOI:** 10.1007/s00262-021-03024-0

**Published:** 2021-08-05

**Authors:** Gilberto Filaci, Daniela Fenoglio, Franco Nolè, Elisa Zanardi, Laura Tomasello, Massimo Aglietta, Gianluca Del Conte, Joan Carles, Rafael Morales-Barrera, Pamela Guglielmini, Giorgio Scagliotti, Alessio Signori, Alessia Parodi, Francesca Kalli, Giuseppina Astone, Francesca Ferrera, Tiziana Altosole, Giuseppina Lamperti, Domenico Criscuolo, Francesco Gianese, Francesco Boccardo

**Affiliations:** 1 Department of Internal Medicine, University of Genoa, Genoa, Italy; 2 Biotherapy Unit, IRCCS Ospedale Policlinico San Martino, Genoa, Italy; 3 Medical Oncology Division of Urogenital and Head and Neck Tumours IEO, European Institute of Oncology IRCCS, Milan, Italy; 4 Academic Unit of Medical Oncology, IRCCS Ospedale Policlinico San Martino, Viale Benedetto XV, 16132 Genoa, Italy; 5 Division of Medical Oncology, Candiolo Cancer Institute, FPO-IRCCS, Candiolo, Italy; 6 Department of Oncology, University of Turin, Turin, Italy; 7 Department of Oncology, IRCCS San Raffaele Hospital, Milan, Italy; 8 Vall d’Hebron Institut d’Oncologia, Barcelona, Spain; 9 Oncology Unit, SS Antonio e Biagio e Cesare Arrigo Hospital, Alessandria, Italy; 10 Department of Oncology, San Luigi Gonzaga Hospital Orbassano and University of Turin, Turin, Italy; 11 Department of Health Science, University of Genoa, Genoa, Italy; 12 Mediolanum Farmaceutici Spa, Milan, Italy

**Keywords:** GX301 cancer vaccine, Telomerase, Prostate cancer, Cancer vaccine schedule, CD8 + T regulatory lymphocytes

## Abstract

Debate is around the optimal immunization regimen for cancer vaccines since too intense vaccination schedules may exhaust reactive lymphocytes. GX301 is a telomerase-based cancer vaccine whose safety and immunological effects were tested in a phase I trial applying an eight administrations schedule. Main objective of this study was to comparatively analyse safety and immunological response to three GX301 regimens in metastatic castration-resistant prostate cancer patients with response/disease stability after docetaxel chemotherapy. This was a multicentre, randomized, parallel-group, open-label trial registered with EudraCT (2014-000095-26) and ClinicalTrials.gov (NCT02293707, 2014). Ninety-eight patients were randomized to receive either eight (regimen 1), four (regimen 2) or two (regimen 3) vaccine administrations. Sixty-three patients were assessable for the primary immunological end-point. Vaccine-specific immune responses were evaluated by intracellular staining for IFN, elispot and cytotoxic assay at 90 and 180 days from baseline. No major side effects were recorded. A 54% overall immune responder rate was observed with 95% of patients showing at least one vaccine-specific immune response. Rate of immunological responders and number of immunizations were proportionally related, suggesting superiority of regimens 1 and 2 over regimen 3. Overall survival did not differ among regimens in both immunological responders and non-responders and was inversely associated (*P* = 0.002) with increase in the number of circulating CD8 + T regulatory cells at 180 days. These data indicate that GX301 cancer vaccine is safe and immunogenic in metastatic castration-resistant prostate cancer patients. Schedules with high number of administrations should be preferred in future studies due to their better immunological outcome.

**Supplementary Information:**

The online version contains supplementary material available at 10.1007/s00262-021-03024-0.

## Introduction

Prostate cancer (PC) is the most common cancer and a leading cause of death from cancer in men [1]. Most of the PC patients with metastatic disease are responsive to androgen deprivation for a limited time and eventually develop castration-resistant disease [2]. Metastatic castration-resistant PC (mCRPC) represents a lethal condition for the great majority of patients, though notable changes occurred in the last 15 years. In 2004, docetaxel was the first drug to demonstrate an overall survival (OS) benefit in mCRPC [3], and in 2010, a comparable advantage was demonstrated for cabazitaxel in patients progressing on docetaxel [4]. Subsequently, abiraterone acetate and enzalutamide were approved for mCRPC in the post-docetaxel [5, 6] and later on in the pre-docetaxel setting [7, 8].

While the introduction of these novel therapies improved the prognosis of mCRPC to a measurable extent in single trials [4–8], their real-life impact is moderate [9–11]. Therefore, new approaches are urgently needed to improve the prognosis of metastatic PC patients, especially when androgen deprivation resistance develops.

PC is potentially an immunogenic tumour [12] so that it could benefit from immuno-stimulating treatments. Several vaccination protocols are under evaluation as anti-cancer therapies [13–15] and sipuleucel-T received FDA and EMA approvals as treatment for PC [16].

Telomerase, the reverse transcriptase responsible for the synthesis, elongation and stability of the telomeric regions of chromosomes [17–20], which is normally expressed by embryonic cells but not by adult somatic cells with a few exceptions, is re-expressed by tumour cells, including PC cells, since essential for tumour immortalization [21–25].

Telomerase is immunogenic, and telomerase-specific T cells were identified in both healthy subjects and cancer patients [26–28], so that telomerase has been proposed as a universal tumour-associated antigen [29].

GX301 is a new telomerase-based cancer vaccine composed of four immunogenic peptides from human telomerase and two complementary adjuvants. The immunogenicity of GX301 was demonstrated in an ex vivo study in which circulating T cell responses to its hTERT peptides were detected in all (100%) of 21 tested subjects [30]. This implies that the four GX301 peptides endow a cumulative epitope pattern wide enough for escaping processes of central tolerance and for inducing telomerase-specific peripheral T cell reactivity in most individuals. A phase I first-in-humans trial, aimed at assessing the safety and immunological effects of GX301 in patients with mCRPC or stage IV renal cancer resistant to conventional treatments, showed evidence of vaccine-specific immunological responses in all patients [31]. In this trial, a fixed vaccination regimen was used, consisting of eight GX301 administrations over a period of 9 weeks [31].

Determining optimal immunization regimens for cancer vaccines is still a problematic issue. Some observations suggest that repeated boosts may exhaust central memory T lymphocytes, which continuously re-populate the compartment of vaccine-specific memory cells [32]. Hence, a too intense vaccination schedule might lead to loss of late immune responses and shortened vaccine efficacy.

We report here the results of a Phase II randomized clinical trial whose main objectives were to compare the immunological response to three GX301 regimens and to extend Phase I findings on GX301 safety in a larger sample of mCRPC patients who achieved response or disease stability after docetaxel chemotherapy.

## Materials and methods

### Study design and patients

This was a multicentre, randomized, parallel-group, open-label trial with blind assessment of the primary end-point. The study was carried out in compliance with the Helsinki Declaration. The protocol was approved by national competent authorities (AIFA and AEMPS, respectively) and the ethics committees of all participating hospitals and was registered with EudraCT (2014-000095-26) and ClinicalTrials.gov (NCT02293707). All patients were required to sign a written informed consent before enrolling into the study.

The primary end-points were safety assessment and evaluation of immunological response defined as the achievement of an immunological score ≥ 3 (see below). Analysis of clinical efficacy was a secondary end-point.

Main eligibility criteria were (a) previously histologically confirmed diagnosis of m CRPC; (b) documented achievement of response or disease stability after docetaxel chemotherapy.

### Treatments

GX301 vaccine is composed of four hTERT peptides (peptides 540–548, 611–626, 672–686, 766–780) and two adjuvants, Montanide ISA-51 VG and imiquimod. Each hTERT peptide was supplied as 625 µg lyophilised powder vials by Bachem AG, Bubendorf, Switzerland. Montanide was supplied as 3 mL vials by Seppic SA, La Garenne Colombes, France. Imiquimod is a medicinal product marketed as single-dose sachets containing 12.5 mg imiquimod as 5% cream (Meda Pharma SpA, Milan, Italy).

Each GX301 administration consisted of four intradermal injections (one for each peptide) given in the abdominal region and followed by topical application of imiquimod. Each intradermal injection consisted of a fixed hTERT peptide dose, 500 μg, reconstituted as a saline solution and mixed with Montanide (1:1) using a standardized disposable device.

The three GX301 regimens consisted of either eight administrations (Regimen 1) on days 1, 3, 5, 7, 14, 21, 35 and 63, four administrations (Regimen 2) on days 1, 14, 35 and 63, or two administrations (Regimen 3) on days 1 and 63 (Supplementary Table 1). Day 1 was the day of randomization.

### Safety assessments

Treatment-emergent adverse events (AEs) were recorded throughout on-study observation. AEs were graded for severity according to Common Terminology Criteria for Adverse Events (CTCAE), version 4.0. AEs that were fatal, life-threatening or requiring/prolonging hospitalization, resulting in significant disability, or otherwise judged as medically important events, were classified as serious.

### Immunological response assessment

Assessment of the immunological efficacy of GX301 regimens was based on the following tests performed on peripheral blood mononuclear cells (PBMC): (1) Peptide-specific ELISPOT assay for the evaluation of frequency of IFNγ–secreting T lymphocytes; (2) Peptide-specific intracellular staining and flow cytometry analysis for evaluation of the frequency of circulating IFNγ–secreting CD4 + and CD8 + T lymphocytes; (3) Peptide-specific cytotoxic assay.

Blood samples for immunological testing were taken at baseline (randomization), day 90 and day 180. Positive test responses found on days 90 and 180 were considered vaccine-related if they were either new (i.e. not detected at baseline) or greater than twice the baseline value. Individual immunological outcomes were scored as the sum of vaccine-related responses, achieving an immunological score ranging 0 to 6 (three tests by two time-points): the immunological success was defined as the achievement of an immunological score ≥ 3.

### Analysis of vaccine-specific IFNγ + T cell frequency by intracellular staining and flow cytometry

The frequencies of IFNγ-secreting CD8 + and CD8- T cells after incubation with the vaccination peptides were evaluated by intracytoplasmic immunofluorescence analyses, as follows. Peripheral blood mononuclear cells (PBMC) (1 × 10^6^ cells), obtained from heparinized peripheral blood by centrifugation on Ficoll gradient and re-suspended in RPMI conditioned by 10% AB serum, were stimulated overnight at 37 °C by a mix of the four GX301 vaccine peptides (5 µg/ml each) in presence of purified anti-human CD28 (clone CD28.2) and anti-CD49d (clone L25) mAbs both at 1 µg/ml concentration (BD). Brefeldine A (BFA, Sigma) (10 µg/ml) was added to samples for the last four hours of incubation. Samples cultured without peptides or stimulated with PMA and ionomycin (Sigma) were considered negative and positive controls, respectively.

Then, washed samples were incubated with vitality dye LIVEDEAD (Molecular Probes, Thermo Fisher) before proceeding with surface staining. The following fluorochrome-conjugated mAbs were used: PE-conjugated anti-human CD8 clone SK1, APC-conjugated anti-human CD3 clone UCHT1 (BD). After surface staining, Cytofix/Cytoperm kit (BD) was used to fix and permeabilize the lymphocytes following the manufacturer’s instructions. The cells were washed in Perm-Wash buffer (BD) and incubated with a FITC-conjugated anti-human IFNγ mAb (BD). Thereafter, the samples were washed in Perm-Wash buffer, re-suspended in FACS Lysing solution (BD) and analysed by a LSR Fortessa X20 flow cytometer (BD) using the FACS DIVA software (BD) v8.1.0. The results were expressed as frequency of IFN-γ producing cells in CD3 + CD8 + or in CD3 + CD8- alive lymphocytes after subtracting the frequency of unstimulated T cells spontaneously producing IFNγ cytokine.

Positive responses were considered those either absent at baseline or greater than twice the baseline showing ≥ 0.1% background positive cells, as suggested for low frequency reactivity [33].

### ELISPOT analyses

In order to detect IFNγ-producing T cells reactive against the GX301 peptides, ELISPOT analyses were performed on freshly isolated PBMC using the Human IFNγ ELISPOT Kit according to the manufacturer’s instructions (BD) and following the indications of international proficiency panels [34]. Briefly, PBMC (2 × 10^5^ cells in X-VIVO medium, Euroclone) were incubated overnight with a mix of the four GX301 vaccine peptides (5 µg/ml each) in the presence of anti-human CD28 and anti-human CD49d mAbs (BD) (both at 1 µg/ml), or with phytohaemagglutinin (PHA-P, MPBIO) at 1 mg/ml, as positive control, or medium alone as negative controls.

Positive responses were considered those either absent at baseline or greater than twice the baseline showing ≥ 10 spots and ≥ 2 × background spot number.

### Cytotoxic assay

Vaccine-specific cytotoxic activity of circulating T lymphocytes was analysed by flow cytometry, as follows [35]. PBMC (10 × 10^6^) were re-suspended in 1 ml of PBS containing CFDA-SE 5 µM (Molecular Probes, Thermo Fisher) for 5 min at room temperature and then washed twice in PBS-1% AB serum at 4 °C. Monocytes were positively sorted from labelled-PBMC by CD14 Micro-Beads human Kit according to the manufacturer’s instructions (Miltenyi) and pulsed or not (1 × 10^5^/well) overnight with a mix of the four GX301 vaccine peptides (5 µg/ml each). The day after, PBMC (2 × 10^6^/ml) were incubated for 6 h at 37 °C with 1 × 10^5^ CFDA-SE-labelled, pulsed or un-pulsed, autologous monocytes as target cells. Thereafter, cells were washed with PBS and re-suspended in 300 µl of PBS added with 5 µl of 7-AAD (BD) before flow cytometer analysis. The samples were analysed by a FACSCanto II flow cytometer (BD) using FACS DIVA software (BD) v 6.1.3.

The percentage of specific lysis was calculated as

$$ {\text{Specific}}\;{\text{ lysis }}\left( \% \right) \, = \frac{{\left( {{\text{CFSE}}^{{{\text{hi}}}} 7 - {\text{AAD}}^{{{\text{pos}}}} } \right)_{{{\text{test }}\;{\text{sample}}}} \left( \% \right) \, - \, \left( {{\text{CFSE}}^{{{\text{hi}}}} 7 - {\text{AAD}}^{{{\text{pos}}}} } \right)_{{{\text{control}}\;{\text{ sample}}}} \left( \% \right)}}{{100 \, - \, \left( {{\text{CFSE}}^{{{\text{hi}}}} 7 - {\text{AAD}}^{{{\text{pos}}}} } \right)_{{{\text{control }}\;{\text{sample}}}} \left( \% \right)}} \times 100 $$

Achieved values were normalized for the percentage of CD3 + CD8 + T cells detected among PBMC. Positive responses were considered those either absent at baseline or greater than twice the baseline showing ≥ 15% of specific lysis.

### Immune phenotyping of peripheral lymphocytes subpopulations

Immune phenotyping of peripheral blood lymphocytes was performed as follows. One hundred µl of washed whole blood, collected in Vacutainers containing tetrasodium EDTA, were incubated with pre-mixed, pre-optimized, multicolour ‘cocktails’ of antibodies within 12 × 75 mm flow cytometry tubes (Lyotube, Becton Dickinson, BD) for 30 min at 4 °C. The cocktails were optimized in two panels to evaluate the frequency of T, B, NK cell subpopulations, CD8 + and CD4 + T regulatory (Treg) cells, CD8 + and CD4 + T cell maturation and activation. The first panel included the following fluorochrome-conjugated monoclonal antibodies (mAbs): BD Horizon V450 (V450)-conjugated anti-human HLA-DR clone L243(G46-6), BD Horizon V450 (V500)-conjugated anti-human CD45 clone 2D1, fluorescein(FITC)-conjugated anti-human CD3 clone UCHT1, allophycocyanin(APC)-conjugated anti-human CD8 clone SK1, APC-H7-conjugated anti-human CD4 clone SK3, phycoerythrin(PE)-conjugated anti-human CD16 + CD56 + clones B73.1 and MY31, PE-Cyanin7(PE-Cy7)-conjugated anti-human CD19 clone SJ25C1. The second panel included the following fluorochrome-conjugated mAbs: V450-conjugated anti-human CD45RA clone HI100, V500-conjugated anti-human CD3 clone UCHT1, Brilliant violet(BV)711-conjugated anti-human CD8 clone SK1, FITC-conjugated anti-human CD127 clone HIL-7R-M21, peridinin-chlorophyll proteins(PerCP)-Cy5.5-conjugated anti-human CCR7 clone 150503, APC-conjugated anti-human CD39 clone TU66, APC-H7-conjugated anti-human CD4 clone SK3, PE-conjugated anti-human CD28 clone CD28.2, PE-Cy7-conjugated anti-human CD25 clone 2A3. Cells were then re-suspended in 100 µl of PBS and 10 µl of 7-AminoactinomycinD (7-AAD, BD) were added as viability staining solution to exclude dead cells. Samples were analysed by a LSR Fortessa X20 flow cytometer (BD) using the FACS DIVA software (BD) v8.1.0.

### Clinical efficacy assessment

Progression-free survival (PFS) and overall survival (OS) were secondary end-points. Assessment of clinical efficacy was based on the evaluation of serum PSA time-course and on the evaluation of disease evolution through clinical examination and imaging analyses repeated at fixed time intervals or at any time if deemed necessary by the local investigators.

### Sample size and statistical analyses

Sample size was estimated under the assumption of the following immunological success rates: regimen A,  ≥ 90%; regimen B, 60–70%; regimen C, ≤ 45%. A sample size of 40 patients per group (total *n*. = 120) had 75% to 99% statistical power to detect the expected differences at both steps of comparison. To compare frequency distribution of variables, contingency analyses were performed by Fisher’s exact test.

PFS and OS were estimated with the Kaplan–Meier method. Data of patients who were lost to on-study observation or follow-up were censored at the time of the last available information. GX301 regimens were compared for PFS and OS using the log-rank test. Association of selected putative prognostic factors (i.e. time to CRPC diagnosis, class of cumulative docetaxel dose and outcome of docetaxel chemotherapy) with PFS or OS was investigated with Cox regression model. All patients who received at least one GX301 administration were included in the analyses.

## Results

### Patient features, number and distribution among the three treatment regimens

Ninety-nine patients were enrolled in the study. One of them withdrew spontaneously: the remaining 98 were randomized into the three regimen groups for receiving either eight (regimen 1, *n* = 32), four (regimen 2, *n* = 33) or two (regimen 3, *n* = 33) vaccine administrations, respectively. All the 98 randomized patients received the vaccine accordingly to the assigned schedule: however, among them, only 63 were assessable for immunological efficacy based on protocol criteria, due to withdrawn from observation before the 180-day time-point of 25 patients.

Baseline patient characteristics are summarized in Table 1.

**Table 1:** Baseline patient features

	Regimen 1 (*n* = 32)	Regimen 2 (*n* = 33)	Regimen 3 (*n* = 33)	All regimens (*n* = 98)
Age (years)*	68.7 (9.8)	70.8 (7.6)	68.3 (8.6)	69.3 (8.7)
Body mass index*	28.1 (5.3)	27.8 (3.0)	28.8 (5.2)	28.2 (4.6)
HLA-A2 + haplotype (*n*.)	11	10	7	28
Time since first PC diagnosis (years)*	5.0 (4.8)	6.8 (5.6)	4.6 (4.5)	5.5 (5.0)
Gleason score**	8.0 (6–10)	8.0 (6–10)	8.0 (6–9)	8.0 (6–10)
Time since CRPC diagnosis (months)*	12.9 (7.4)	13.1 (7.9)	15.0 (10.2)	13.7 (8.6)
Pre-docetaxel abiraterone or enzalutamide (*n*.)	3	4	2	9 (9.2%)
Cumulative docetaxel dose				
300–525 mg/m^2^ (*n*.)	10	10	13	33 (33.7%)
526–825 mg/m^2^ (*n*.)	21	21	20	62 (63.3%)
> 825 mg/m^2^ (*n*.)	1	2	0	3 (3.1%)
Time since last docetaxel infusion (weeks)*	10.8 (5.9)	12.5 (9.1)	12.8 (17.2)	12.1 (11.7)
Docetaxel outcome				
Response (*n*.)	26	20	30	76 (77.6%)
Disease stability (*n*.)	6	13	3	22 (22.4%)
Metastatic sites				
Skeletal only (*n*.)	8	13	13	34 (34.7%)
Soft tissue (nodal, visceral) only (*n*.)	6	5	5	16 (16.3%)
Bone and soft tissue (*n*.)	18	14	15	47 (48.0%)
Serum testosterone < 1.7 nmol/L (*n*.)	30	33	33	96 (98.0%)
ECOG performance status 0/1 (*n*.)	24/8	24/9	20/13	68/30

*Mean (SD); **Median (range)

### Safety assessment

Safety was analysed in all 98 patients who received at least one vaccine administration. Among AEs, panniculitis-like local inflammation at the site of vaccine administration was common to all patients. The frequency of inflammatory reaction at injection sites increased, as expected, with the number of vaccine administrations. Overall, SAEs were rare and mostly unrelated to GX301 vaccination. In particular, only one fatal event was registered due to the onset of a second neoplasia (glioblastoma multiforme). Table 2 summarizes serious non-fatal adverse events: a total of eight SAEs was observed in two, two and one patients in regimen 1, regimen 2 and regimen 3, respectively.

**Table 2:** Summary of non-fatal treatment-emergent SAEs

SAE type	Regimen 1 *n*. (%)	Regimen 2 *n*. (%)	Regimen 3 *n*. (%)
Loss of consciousness			1 (3)
Motor dysfunction	1 (3)		
Anaemia		1 (3)	
Esophagitis		1 (3)	
Gastritis		1(3)	
Systemic inflammatory syndrome		1 (3)	
Infections	1 (3)		
Neoplasm (bladder cancer)		1 (3)	
Total subjects with non-fatal treatment-emergent SAEs	2 (6.3)	2 (6.1)	1 (3)

Importantly, no events relative to induction of severe lymphopenia after vaccination were registered in our series (not shown).

Moreover, no onset of autoimmunity-related clinical signs or of autoantibodies was observed.

### Immunological response

**Fig. 1:**
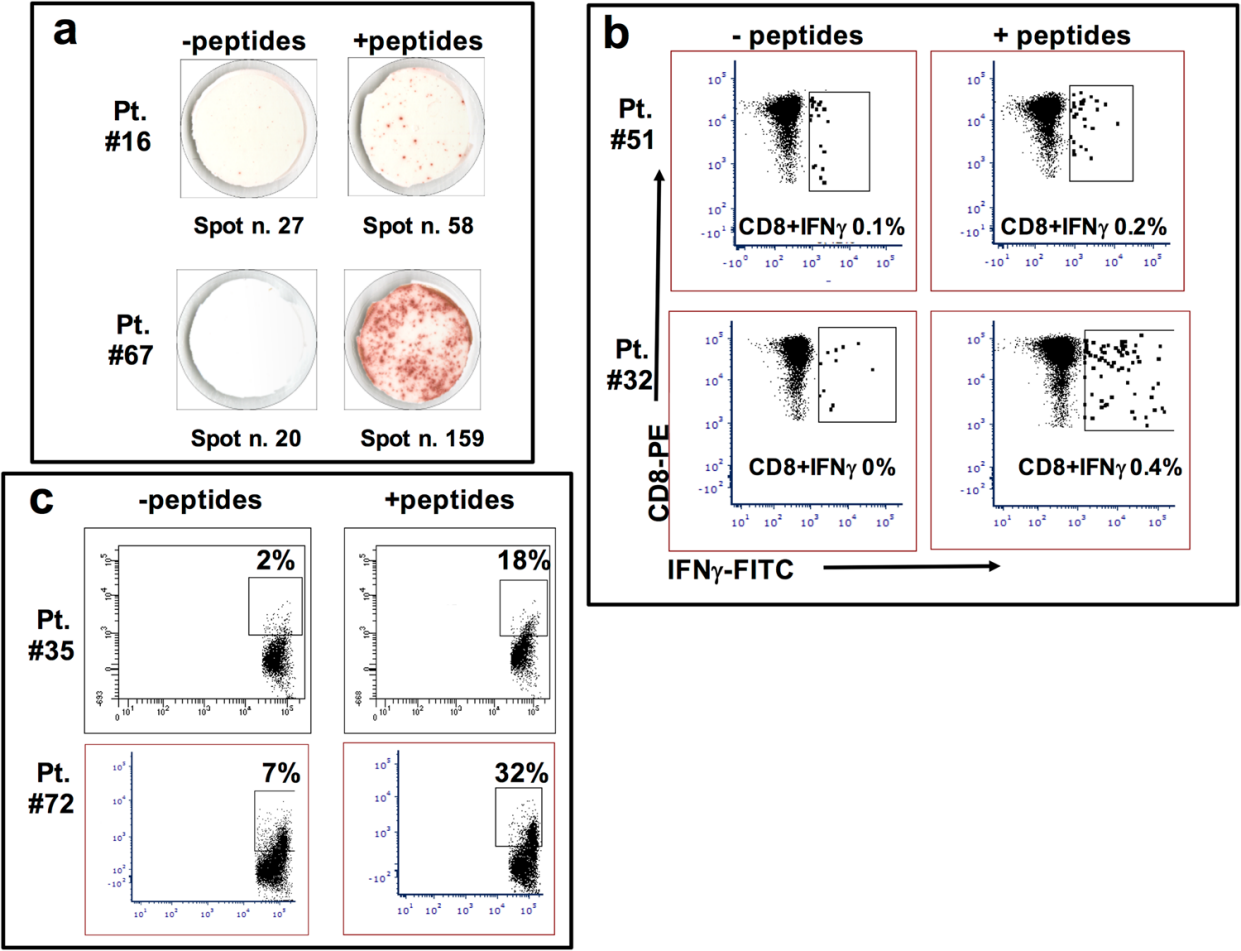
Representative examples of performed immunological analyses. **a** ELISPOT assays performed after 180 days from vaccination on PBMC from patient #16 (low response, upper panels) and after 90 days from vaccination on PBMC from patient #67 (high response, lower panels); **b** intracellular cytokine staining of CD8-IFNγ + and CD8 + IFNγ + circulating T cells specific for GX301 peptides after 180 days from vaccination in blood sample derived from patient #51 (low response, upper panels) and after 90 days from vaccination in blood sample derived from patient #32 (high response, lower panels); **c** GX301 peptides specific cytotoxicity assays performed after 90 days from the first immunization with PBMC of patient #35 (low response, upper panels) and patient #72 (high response, lower panels) against autologous monocytes pulsed (right panels) or not (left panels) with the four GX301 peptides

Sixty-three patients were assessable for the immunological outcome (*n* = 20, *n* = 24 and *n* = 19 for regimen 1, regimen 2 and regimen 3, respectively). Representative analyses for each type of immunological tests are shown in Fig. 1.

Sixty out of 63 (95%) immunized patients who completed the vaccination protocol showed at least one positive response at one of the tests performed on days 90 and 180 after the first immunization. The only three patients who did not show any vaccine-specific immune response belonged to the regimen 3 (Table 3).

**Table 3:** Immunological score and responder rate among assessable GX301 treated patients

Immunological score* (*n*.)	Regimen 1 (*n* = 20)	Regimen 2 (*n* = 24)	Regimen 3 (*n* = 19)	All regimens (*n* = 63)
0	0	0	3	3 (4.8%)
1	4	3	4	11 (17.5%)
2	3	8	4	15 (23.8%)
3	12	11	4	27 (42.9%)
4	1	1	4	6 (9.5%)
5	0	1	0	1 (1.6%)
6	0	0	0	0
Immunological responders** (score ≥ 3), *n*. (%)	13 (65)	13 (54.2)	8 (42.1)	34 (54.0)

*The immunological score was the sum of positive responses observed at the immunological tests performed on days 90 and 180 after vaccination

**As per protocol criteria, immunological responders were patients achieving an immunological score ≥ 3

Responders to vaccination, as per protocol criteria, ranged from 42 to 65% of patients with a proportional relationship between rates of immunological responders and number of immunizations administered by each regimen (Table 3).

Since one of the four immunogenic peptides (hTERT_540–548_) included in the GX301 vaccine is restricted by the HLA-A_2_ allele, we compared the responder rates between the HLA-A_2_ positive and negative patients and no differences were observed (not shown).

**Fig. 2:**
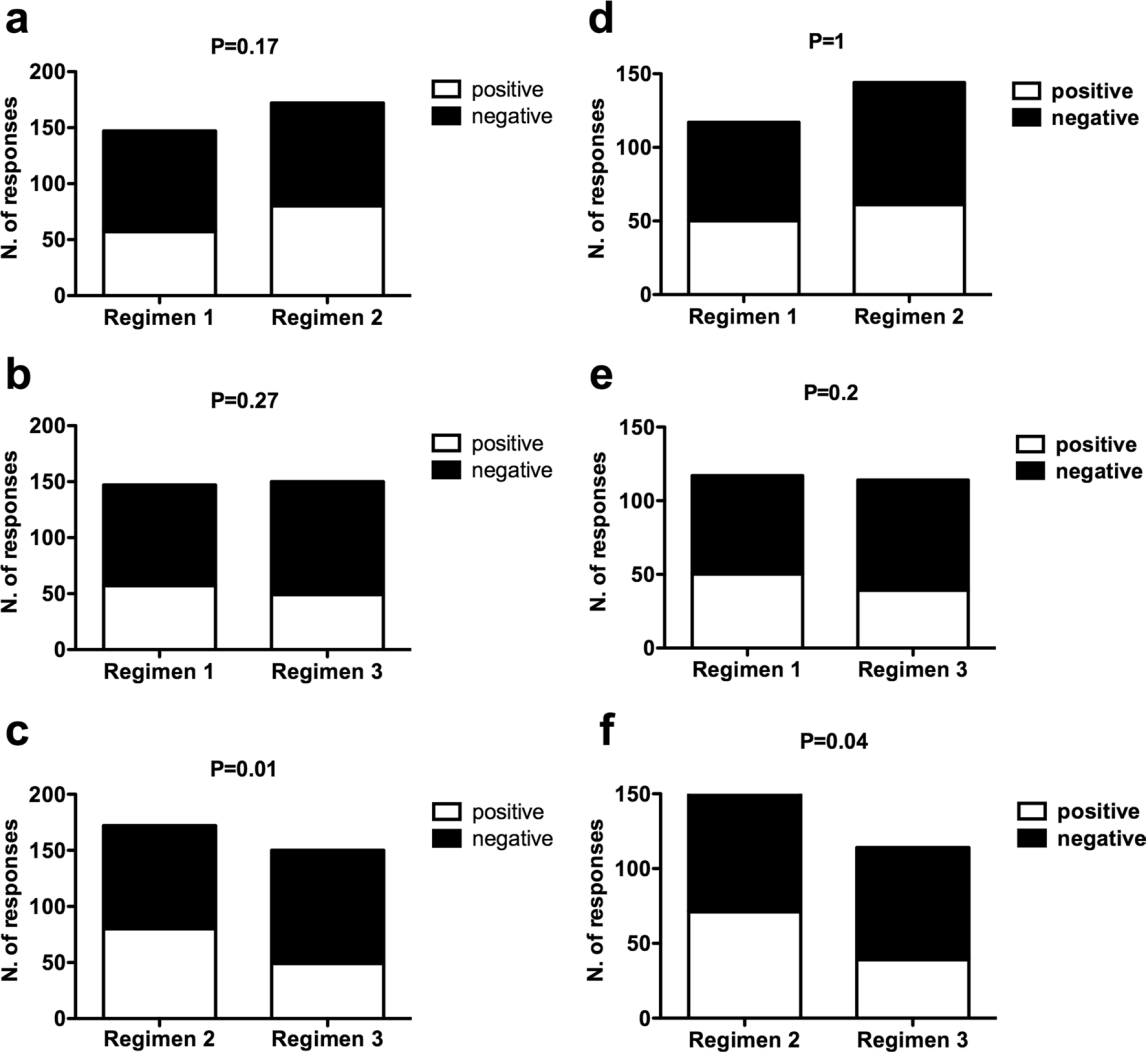
Comparison of response rates among the different regimens. Contingency analyses comparing the number of positive and negative immunological responses between either regimen 1 and regimen 2 (**a** and **d**), regimen 1 and regimen 3 (**b** and **e**), or regimen 2 and regimen 3 (**c** and **f**) among either the overall 98 patient series (**a**–**c**) or the 63 patients assessable for the immunological outcome (**d**–**f**). Concerning the overall 98 patient series (**a**–**c**), the total numbers of performed tests were 117, 144 and 114 for regimens 1, 2 and 3, respectively; concerning the group of 63 patients assessable for the immunological outcome (**d**–**f**), the total numbers of performed tests were 147, 172 and 150 for regimens 1, 2 and 3, respectively

Interestingly, taking into consideration the total number of positive and negative responses to the six immunological tests performed at days 90 and 180 in either the total patient population receiving the vaccine (*n*. 98 patients) or the immunological assessable patient population (63 patients), the comparison of the rates of positive responses at any of the six immunological tests among the different regimens showed a significant difference between regimen 2 and regimen 3, while no differences were observed between either regimen 1 and regimen 3 or regimen 1 and regimen 2 (Fig. 2).

### Analyses of T cell subpopulations

The circulating frequencies and absolute numbers of different T cell subsets were assessed at baseline and after 90 and 180 days from baseline. T cell subsets to be analysed were selected for (a) maturation stage, in terms of CCR7 + CD45RA + naïve, CCR7 + CD45RA- central memory (CM), CCR7 + CD45RA- effector memory (EM), and CCR7 + CD45RA + terminal effector memory cells (TEM), and b) regulatory commitment, in terms of both CD4 + CD127-CD25hi and CD8 + CD28-CD127loCD39 + Treg, respectively.

**Fig. 3:**
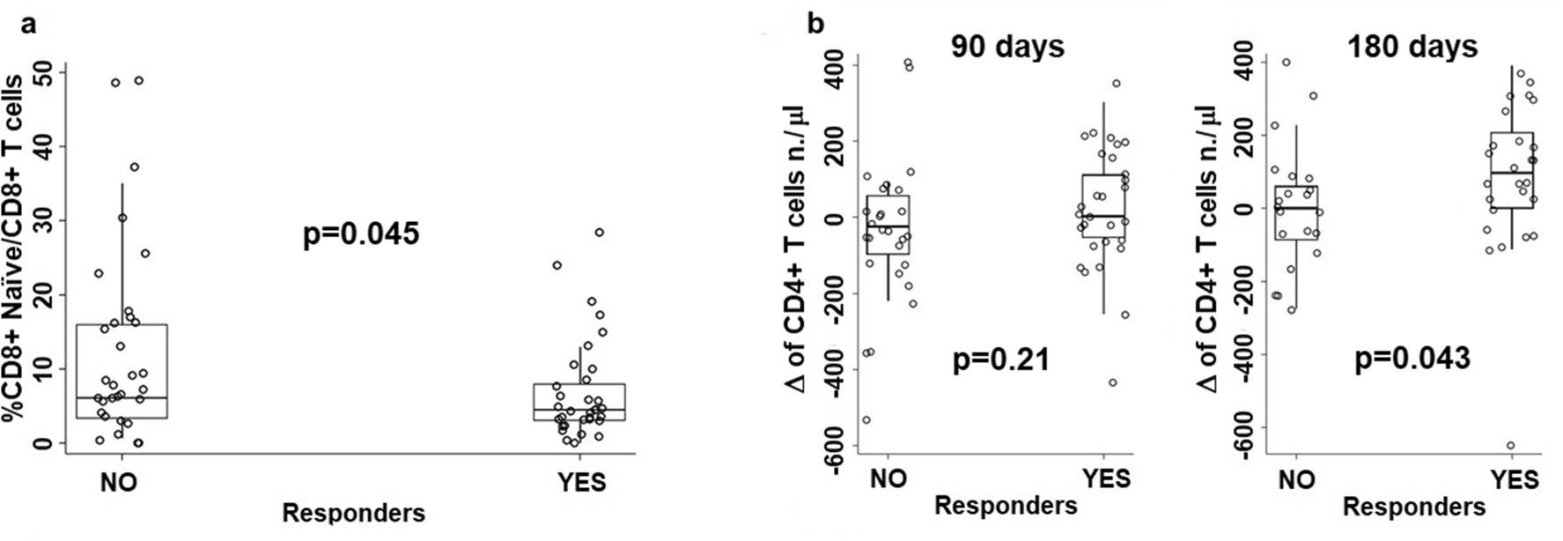
Comparisons of T cell subset frequency or number between immunologically responder and non-responder patients relative to: **a** comparison between immunologically responder and non-responder patients relative to the frequency of naïve CD8 + T cells at baseline; **b** comparisons between immunologically responder and non-responder patients concerning the differences between day 90 or day 180 values and baseline relative to the absolute number of circulating CD4 T cells

The comparison of circulating T cell subset frequencies or absolute numbers between immunologically responder and non-responder patients showed that at baseline the only difference concerned the frequency of naïve CD8 + T cells, that was lower in responders than in non-responder patients (Fig. 3a).

In order to have a picture on the dynamics of T cell subset frequencies and absolute numbers upon GX301 vaccination, the differences (Δ) were calculated between values at day 90 or at day 180 and values at baseline; then, such differences were compared between responders and non-responders. This analysis showed that responders had a significantly higher increase in absolute number of circulating CD4 + T cells at day 180 than non-responders (Fig. 3b).

### Clinical outcome

**Fig. 4:**
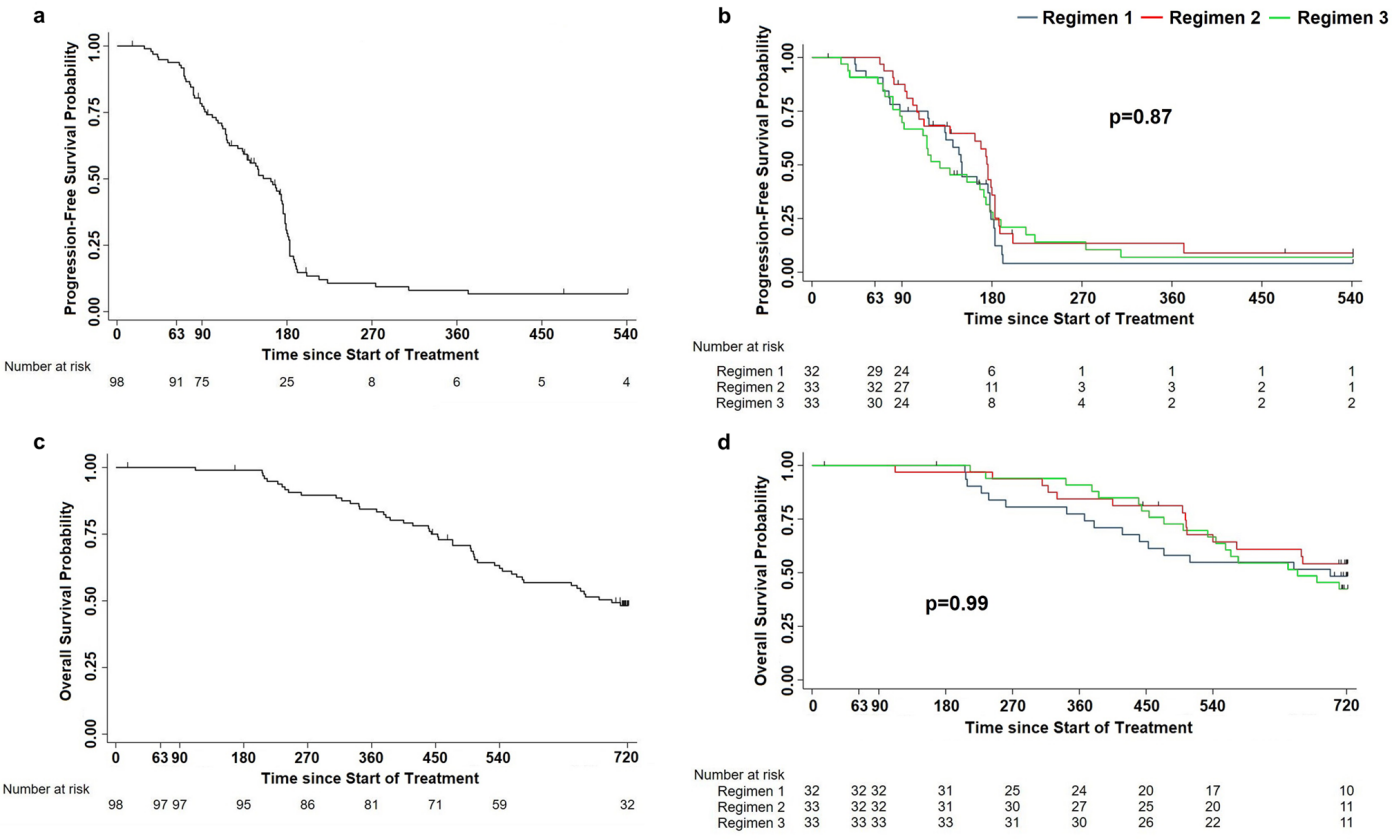
Progression-free survival and overall survival in the overall study population or according to GX301 regimens. **a** Progression-free survival in the overall study population. Number of censored cases before the end of observation: 16; number of progressions by day 540: 82; time to progression: 163 days (IQR: 95–183); estimated Progression-free survival: 77.3% at day 90, 29.5% at day 180, 10.7% at day 270. **b** Progression-free survival according to GX301 regimens. Median times to progression (IQR) for regimen 1, regimen 2 and regimen 3 were, respectively, 150 days (102–179), 176 days (105–187) and 128 days (88–181). **c** Overall survival in the overall study population. Number of censored cases before the end of observation: 49; number of deaths by day 720: 49; median time to death (IQR): 698 (452-not reached); estimated survival: 99% at day 180, 84% at day 360, 62% at day 540, 48% at day 720. **d** Overall survival according to GX301 regimens. Numbers of deaths by day 720 were for regimen 1, regimen 2 and regimen 3 were 16, 14 and 19, respectively; median times to death (IQR) for regimen 1, regimen 2 and regimen 3 were, respectively, 698 (367-not reached), not determined (503-not reached), 654 (474-not reached)

Fifty per cent of patients underwent disease progression within day 163 and 75% within day 183 (Fig. 4a), with no statistically significant or trend differences among GX301 regimens (Fig. 4b).

Post-study follow-up was completed by 95% of patients. OS was 62% at 18 months and 48% at 24 months (Fig. 4c) with no statistically significant differences among GX301 regimens (Fig. 4d).

**Fig. 5:**
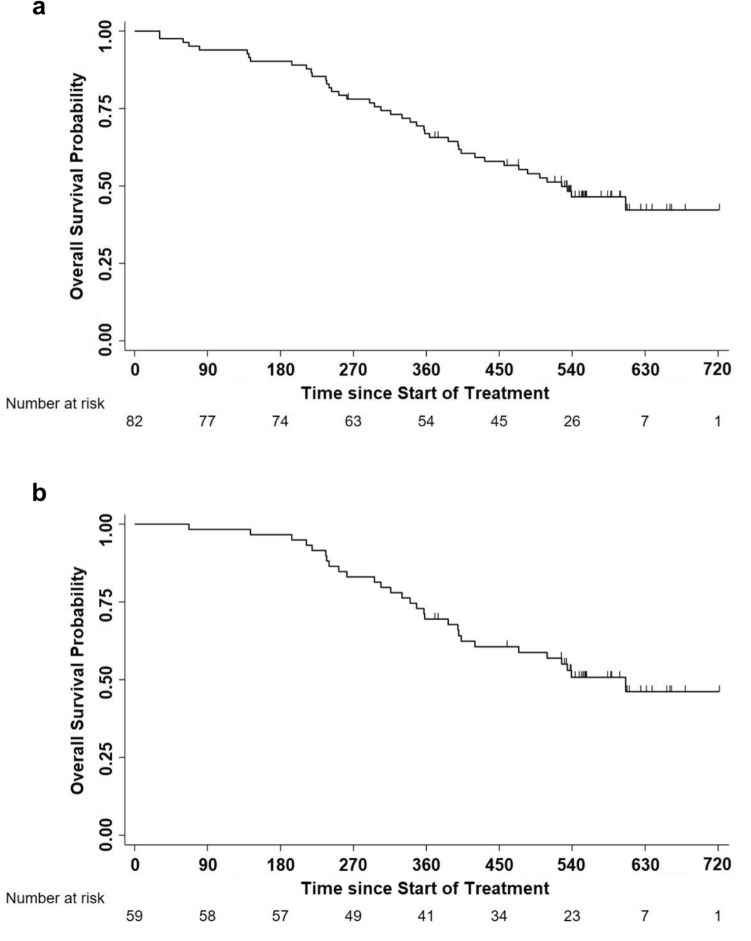
Overall survival in patients with documented disease progression. **a** Overall survival in 82 patients with documented disease progression treated with GX301 vaccine. **b** Overall survival in 59 patients with documented disease progression treated with GX301 vaccine and then with either abiraterone acetate, enzalutamide, or cabazitaxel (used singularly or in sequential combination)

In order to have a rough estimate of clinical efficacy of the vaccine, the analysis of survival following disease progression was calculated for patients with documented disease progression (*n* = 82 out of 98 enrolled patients). Median survival of patients progressing after vaccine administration was 17.3 months (Fig. 5a). This value increases to 19.9 months limiting the analysis to the 59 patients who were treated at progression with either abiraterone acetate, enzalutamide or cabazitaxel (used alone or in sequential combinations) (Fig. 5b).

### Relationship between immunological parameters and clinical outcome

**Fig. 6:**
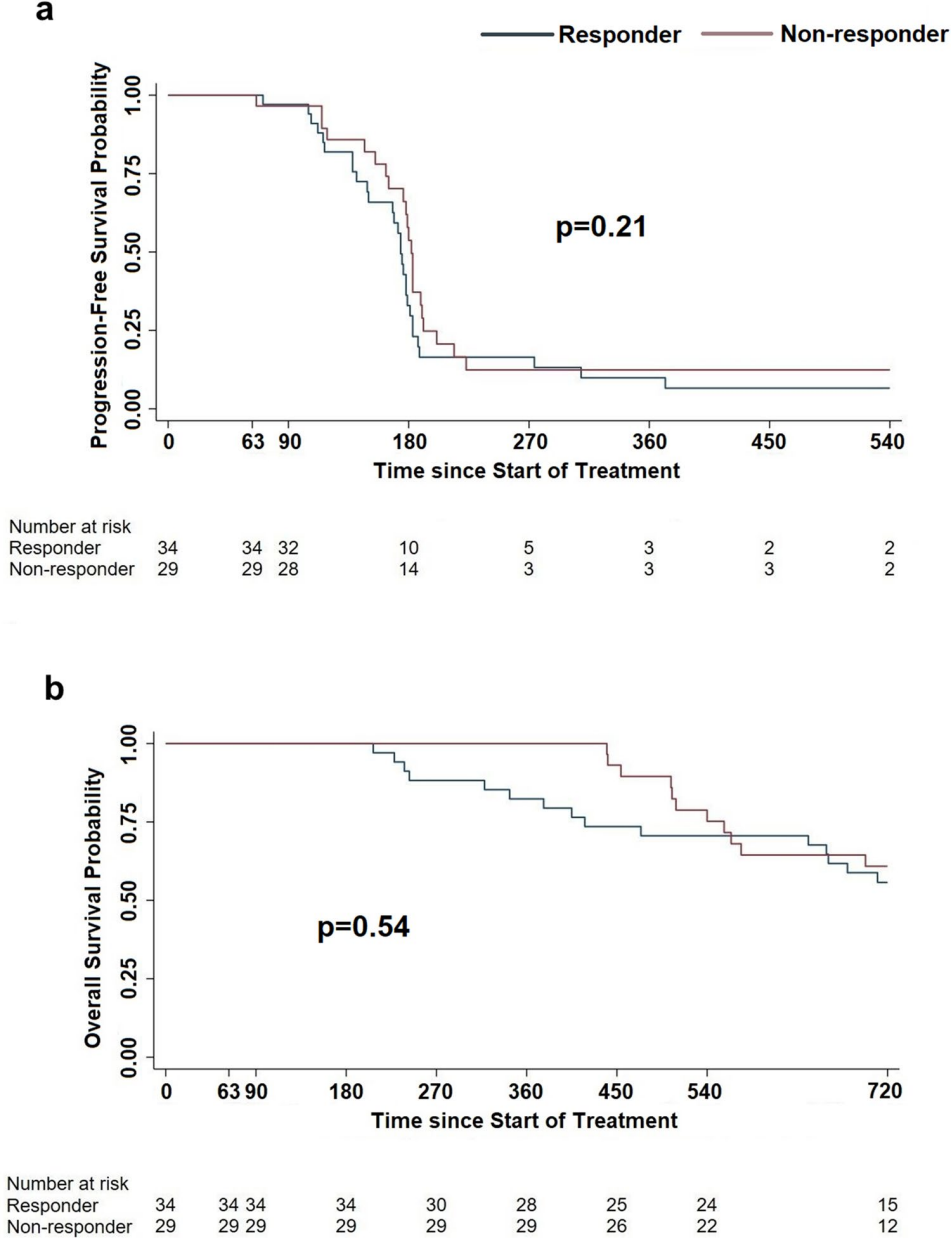
Progression-free survival (**a**) and overall survival (bb) in immunologically responder vs non-responder patients. **a** Median time to progression for responder and non-responder patients were 174 days and 182 days, respectively; **b** Median time to death was not reached in either group

In order to investigate on the possible association between immunological response to GX301 vaccine and clinical outcome, PFS and OS were compared between responders and non-responders, irrespective of the assigned regimen. Figure 6a, b shows that no significant differences were observed between the two groups.

**Fig. 7:**
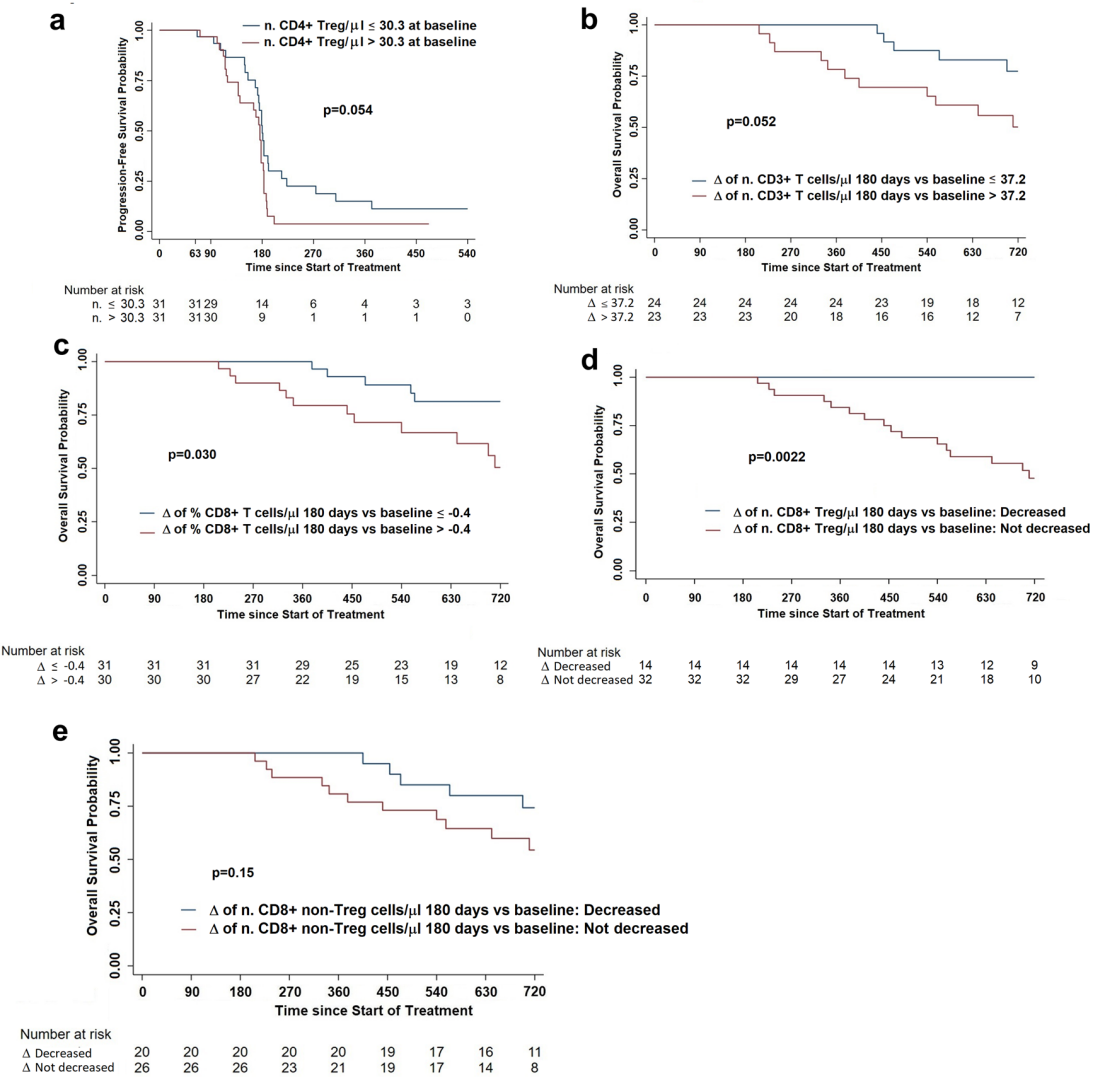
Progression-free survival (**a**) and Overall survival according to the level or dynamics of different T cell subsets. **a** Progression-free survival according to the circulating absolute number of CD4 + Treg at baseline; **b** Overall survival according to a 180 days versus baseline Δ of circulating CD3 + T cell number/μl ≤ (blue line) or > (red line) 37.2; **c** Overall survival according to a 180 days versus baseline Δ of circulating CD8 + T cell frequency ≤ (blue line) or > (red line) − 0.4; **d** Overall survival according to a 180 days versus baseline Δ of circulating CD8 + Treg number/μl decreased (blue line) or not-decreased (red line);** e** Overall survival according to a 180 days versus baseline Δ of circulating CD8 + non-Treg number/μl decreased (blue line) or not-decreased (red line)

Then, we wondered whether the level or dynamics of some T cell subsets could be predictive of clinical outcome. Hence, we found that baseline absolute number of CD4 + Treg impacted on PFS since patients with baseline number < 30.3 CD4 + Treg/μl had a lower risk of progression than patients with baseline number > 30.3 CD4 + Treg/μl (Fig. 7a). Concerning OS, we observed that patients who had a day 180 vs baseline Δ < 37.2 of CD3 + T cell number/μl and < − 0.4 of CD8 + T cell percentage had a more prolonged survival than patients with Δ values > 37.2 CD3 + T cell number/μl and > − 0.4 CD8 + T cell percentage, respectively (Fig. 7b, c).

Interestingly, a decreased number at day 180 of CD8 + Treg (identified as shown in Supplementary Fig. 1) was associated with a better prognosis (Fig. 7d), a phenomenon non-dependent on the trend of the non-Treg CD8 + T cell subpopulation (Fig. 7e).

## Discussion

How the regimen of a cancer vaccine may impact on its efficacy? High number of administrations may have a bi-faceted effect, either boosting the immunization or exhausting the memory immune response [32]. Hence, this trial was specifically designed to comparatively analyse, other than safety, the immunological response to three different GX301 cancer vaccine schedules in a cohort of mCRPC patients.

GX301 vaccination proved to be remarkably safe at all tested regimens. No serious or Grade ≥ 3 AEs were considered to be treatment-related. Laboratory tests aimed at detecting possible autoimmune reactions yielded essentially negative findings. The most represented AE was the skin inflammatory reaction at GX301 injection sites often associated with flu-like systemic symptoms.

Concerning the immunological outcomes, GX301 proved to be effective in inducing some immunological response in 95% of patients (100% with the more intense regimens). Moreover, immunological success, as per protocol criteria, was achieved by 65% of patients with the most intense regimen. These results, confirming in a wider series those achieved in a previous small phase I trial [31], support that immune tolerance does not remarkably affect responses against telomerase, although it is an endogenous antigen. This is not surprising since telomerase is stably expressed only during the foetal life, at a time when the immune system is not mature yet. After birth, cells repress the telomerase gene, which is only fleetingly expressed by stem cells or by actively replicating cells [36]. Hence, when tumour cells appear in the organism and constitutively re-express telomerase, T lymphocytes recognize it as a new antigen and mount a specific immune response, a capacity shared by the immune T cells of both cancer patients and healthy subjects (30). Interestingly, immunological responders showed lower frequency of naïve CD8 + T cells at baseline and higher number of CD4 + T cells at day 180 from vaccination than non-responders. These findings imply that a pre-vaccination increased repertoire of effector/effector memory CD8 + T lymphocytes and achievement upon vaccination of a robust CD4 + T cell response are essential requirements for an effective anti-cancer vaccination.

The weaknesses of this study are the actual sample size of treated patients (*n* = 98) fell short of the planned one (*n* = 120) and that the immunological outcomes could not be assessed in 35 patients, so that the assessable sample was reduced to 63 patients: hence, between-regimen differences in responder rates were smaller than assumed in the protocol estimation of the sample size, not reaching conventional statistical significance. Notwithstanding this fact, the results indicate a superiority of the 8- and 4-administration regimens over the 2-administration scheme suggesting that for GX301 cancer vaccine, repeated administrations are necessary for inducing effective immunization. Interestingly, part of the detected responses was long-lasting (6 months after the first vaccine administration) suggesting the capacity of GX301 vaccine to induce memory T cell responses.

In the present study, PFS and OS did not appear to be related to immunological outcomes. However, the trial was not powered to detect differences between vaccination regimens in PFS or OS. In order to have indications on the clinical efficacy of GX301 vaccine, we calculated the median OS from progression in all patients with documented progression (17.3 months), as well as in patients with documented progression treated thereafter with either abiraterone acetate, enzalutamide and cabazitaxel (19.9 months). Interestingly, these OS values were non-inferior to those of patients failing after front-line docetaxel, that were reported to be 15.8, 18.4 and 15.1 months after treatment with abiraterone acetate [5], enzalutamide [6], or cabazitaxel [4], respectively. These findings are not sufficient per se to suggest a beneficial effect of the vaccination on patients survival; however, they allow to rule out any putative detrimental effect in this regard. Moreover, the immune efficacy shown by GX301 in our study rules out that previous docetaxel treatment might have blunted the immunological response to the vaccine, confirming the data of other recent reports [37–42].

Interesting insights came from the evaluation of the dynamics of circulating Treg subsets. We observed that PFS after GX301 vaccination was inversely dependent on the frequency of CD4 + Treg at baseline, reminiscent of what already observed in experimental animal models [43]. Moreover, the circulating number of CD8 + CD28-CD127-CD39 + Treg, a CD8 + Treg subset that heavily infiltrates human cancer [44, 45], showed a highly significant predicting value since reduced (with respect to baseline) levels of these cells at 180 days after GX301 vaccination predisposed patients to a prolonged survival. The setting of our study cannot permit to differentiate whether the predicting value of circulating CD8 + CD28-CD127-CD39 + Treg number relates to the effects of the vaccination or to the chemotherapy (or to their combination). However, these findings, suggesting a shift from effector to regulatory T cell functions, support the opportunity to constitutively associate cancer vaccines with the administration of agents able to counteract Treg activity (i.e. specific immune checkpoint blockers).

## Conclusions

The results of our study show that (a) GX301 cancer vaccine is substantially safe and immunogenic; (b) higher numbers of administrations provide a better immunological response than lower numbers; (c) the median OS from disease progression is promising enough to suggest the opportunity to further testing GX301 in m CRPC; (d) the dynamics in the circulation of specific T cell subsets, in particular of Treg, may have a prognostic value. This latter point, providing a possible mechanism explaining the poor efficacy of cancer vaccines, except sipuleucel, in PC, opens a perspective for the combination of GX301, administered with a four or eight administration schedules, with immune-check point inhibitors in the treatment of PC.

## Supplementary Information

Below is the link to the electronic supplementary material.

Supplementary file1 (PDF 109 KB)

## Data Availability

The data presented in this study are available in this article (and supplementary material). Raw data can be provided per request.
