# Supplementary material for: Telomerase-based GX301 cancer vaccine in patients with metastatic castration-resistant prostate cancer: a randomized phase II trial
Source: Cancer Immunol Immunother. 2021 Aug 5;70(12):3679–92. doi: 10.1007/s00262-021-03024-0 (PMC8571235; doi:10.1007/s00262-021-03024-0)
Supplement: Supplementary file 1 — Supplementary file1 (PDF 109 KB) [file 262_2021_3024_MOESM1_ESM.pdf]

Supplementary Table 1. GX301 administration regimens adopted in the study.

Regimens	Days of vaccine administration							
	**Day 1**	**Day 3**	**Day 5**	**Day 7**	**Day 14**	**Day 21**	**Day 35**	**Day 63**
Regimen 1 (8 administrations)	X	X	X	X	X	X	X	X
Regimen 2 (4 administrations)	X				X		X	X
Regimen 3 (2 administrations)	X							X

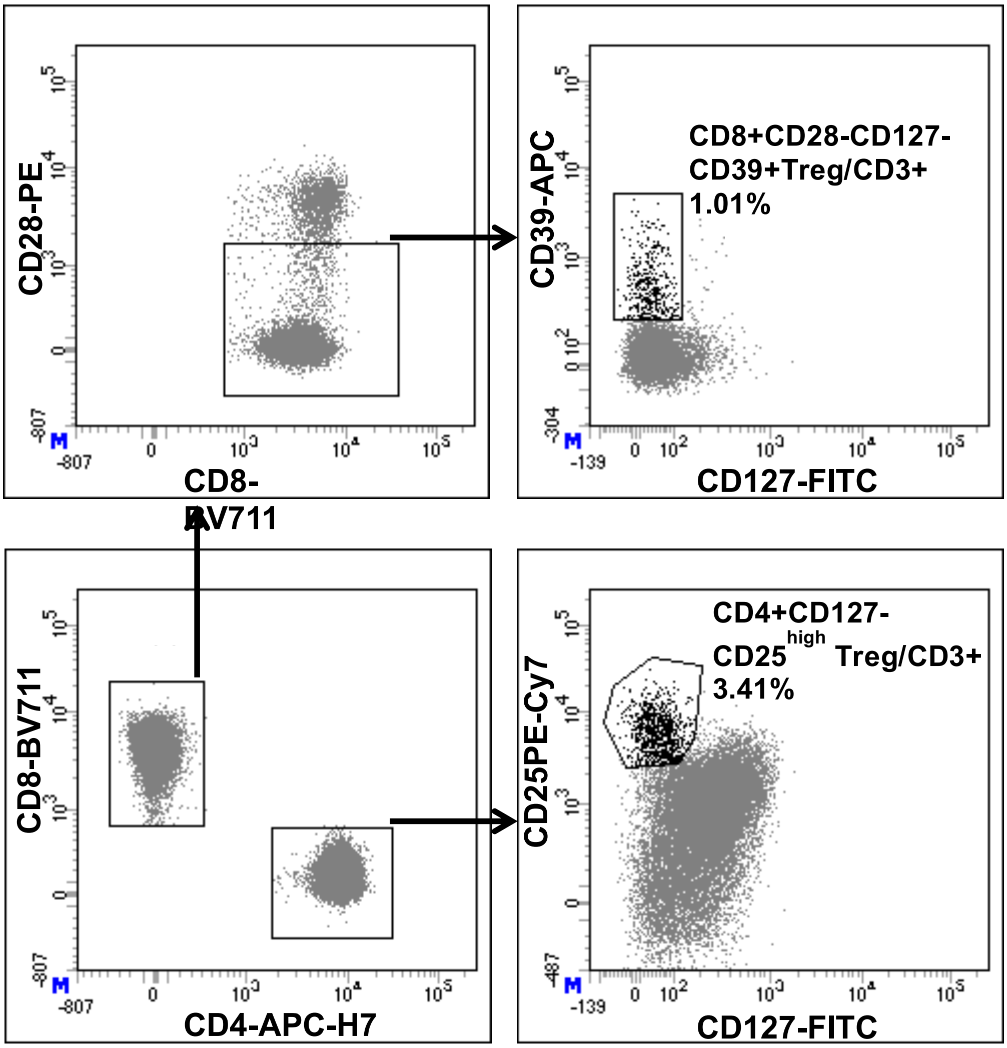

Supplementary Fig. 1. Representative example of gating strategy for the identification of CD4+ CD127-CD25high and CD8+CD28-CD127-CD39+ T regulatory lymphocytes from peripheral blood of patient #14 after 180 days from vaccination.
